# Advances in the research of the mechanism of secondary resistance to imatinib in gastrointestinal stromal tumors

**DOI:** 10.3389/fonc.2022.933248

**Published:** 2022-09-06

**Authors:** Xiangchen Hu, Zhe Wang, Peng Su, Qiqi Zhang, Youwei Kou

**Affiliations:** ^1^ Department of General Surgery, Shengjing Hospital of China Medical University, Shenyang, China; ^2^ Department of Pathology, Shengjing Hospital of China Medical University, Shenyang, China; ^3^ Medical Research Center, Shengjing Hospital of China Medical University, Shenyang, China

**Keywords:** gastrointestinal stromal tumor, imatinib, secondary imatinib resistance, drug-resistance mechanism, therapeutic targets

## Abstract

Gastrointestinal stromal tumors (GISTs) are the most common mesenchymal tumors of the gastrointestinal tract. At present, surgery is the first-line treatment for primary resectable GISTs; however, the recurrence rate is high. Imatinib mesylate (IM) is an effective first-line drug used for the treatment of unresectable or metastatic recurrent GISTs. More than 80% of patients with GISTs show significantly improved 5-year survival after treatment; however, approximately 50% of patients develop drug resistance after 2 years of IM treatment. Therefore, an in-depth research is urgently needed to reveal the mechanisms of secondary resistance to IM in patients with GISTs and to develop new therapeutic targets and regimens to improve their long-term prognoses. In this review, research on the mechanisms of secondary resistance to IM conducted in the last 5 years is discussed and summarized from the aspects of abnormal energy metabolism, gene mutations, non-coding RNA, and key proteins. Studies have shown that different drug-resistance mechanism networks are closely linked and interconnected. However, the influence of these drug-resistance mechanisms has not been compared. The combined inhibition of drug-resistance mechanisms with IM therapy and the combined inhibition of multiple drug-resistance mechanisms are expected to become new therapeutic options in the treatment of GISTs. In addition, implementing individualized therapies based on the identification of resistance mechanisms will provide new adjuvant treatment options for patients with IM-resistant GISTs, thereby delaying the progression of GISTs. Previous studies provide theoretical support for solving the problems of drug-resistance mechanisms. However, most studies on drug-resistance mechanisms are still in the research stage. Further clinical studies are needed to confirm the safety and efficacy of the inhibition of drug-resistance mechanisms as a potential therapeutic target.

## 1 Introduction

Gastrointestinal stromal tumors (GISTs) are the most common mesenchymal tumors of the digestive tract, originating from gastrointestinal pacemaker cells (interstitial cells of Cajal, ICC) or related stem cells (1, 2). GISTs are typically driven by mutations of the receptor tyrosine kinase oncogene (C-KIT) or the platelet-derived growth factor receptor α (*PDGFR*α), which account for more than 80% and 5%–10% of all cases of GIST, respectively (3–5). GISTs without *KIT* or *PDGFR*α mutations are known as wild-type GISTs (WT-GISTs), which account for 10%–15% of all cases of adult GISTs and up to 85% of all cases of pediatric GISTs (6–8). In this category, 20%–40% are characterized by the loss of succinate dehydrogenase complex (SDH-deficient GISTs), approximately 15% carry *BRAF*/*RAS* or *NF1* mutations, and the remainder is referred to as KIT/PDGFRA/SDH/RAS-P WT-GISTs (or quadruple WT-GISTs) (9, 10). A careful examination for germline mutations is of great significance for all patients with WT-GISTs (11). Analyses conducted using tissue microarrays have shown that the *DOG1* gene is relatively specifically expressed in GISTs, regardless of the *KIT* or *PDGFRA* mutation status (12). A monoclonal antibody against *DOG1* has been proven to be a highly sensitive specific marker for the diagnosis of GISTs, and its sensitivity is higher than that of *KIT* (13).

**Figure 1:**
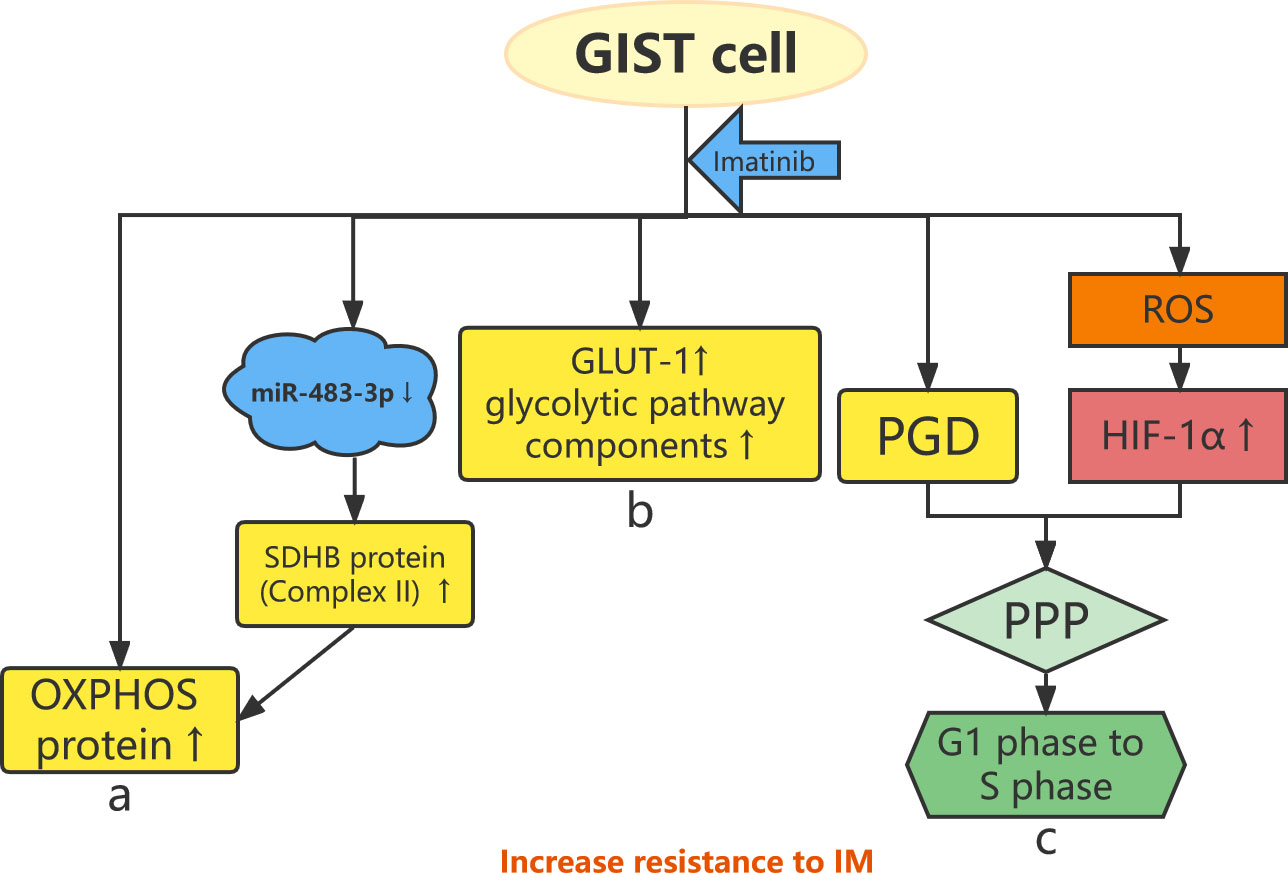
Abnormal energy metabolism and resistance to imatinib. **(A)** OXPHOS protein expression is increased in IM-resistant GIST cells, and IM specifically increases the expression of complex II (SDHB) protein by downregulating miR-483-3p. **(B)** GLUT-1 and glycolytic pathway components increase in IM-resistant GIST cells. **(C)** The HIF-1α–PGD–PPP axis and IM-induced ROS stimulate GIST cells from the G1 phase to the S phase, leading to drug resistance.

Imatinib mesylate (IM) is a selective tyrosine kinase inhibitor (TKI) that targets *KIT* and *PDGFR*α for the treatment of unresectable or metastatic GISTs, which significantly improves the 5-year survival of patients (14, 15). The efficacy of IM varies among different *KIT* and *PDGFRA* mutation types, depending on the exons involved (16, 17). Approximately 14% of patients with GISTs are initially resistant to IM (18), whereas approximately 50% of patients develop resistance after 2 years of treatment, the so-called secondary resistance to IM (19). Therefore, it is important to clarify the mechanisms of secondary resistance to IM through research and develop new therapeutic targets and regimens to improve the long-term prognoses of patients with GISTs. A recent study demonstrated that IM specifically increases the expression of the complex II (SDHB) protein in oxidative phosphorylation (OXPHOS) proteins by downregulating miR-483-3p (**Figure 1A**). This study demonstrated the molecular mechanism of increased OXPHOS protein expression induced by IM and confirmed the biological role of miR-483-3p in regulating energy metabolism after IM treatment (20).This review will focus on the discussion and summary of research on the mechanisms of secondary resistance to IM conducted in the last 5 years from the aspects of abnormal gene mutation, energy metabolism, non-coding RNA, and key proteins.

## 2 Mechanisms of secondary resistance to imatinib

### 2.1 Gene mutation and resistance to imatinib

Secondary *KIT* and *PDGFRA* mutations are the main causes of secondary resistance to IM in non–wild-type GIST (21, 22). In most cases of GIST, secondary KIT mutations reactivate *KIT* downstream signaling pathways, such as the PI3K/AKT/mTOR pathway, and continue to drive GIST proliferation and survival, leading to acquired IM resistance (23–27). KIT T670I is one of the most common types of secondary KIT mutations (24). Cassier et al. found that *PDGFRA* exon 18 D842V gene subtype mutation is associated with primary resistance to IM (16). Secondary *PDGFRA* mutations are less common in IM-resistant GISTs than secondary *KIT* mutations (28, 29). Secondary *KIT* mutations or *PDGFRA* mutations do not occur in wild-type IM-resistant GISTs (30).

In a previous study of 210 Chinese patients with IM-resistant GIST who underwent next-generation sequencing for the identification and characterization of secondary *KIT* mutations, the results showed that 63.81% of the patients had mutations on exon 13, 4.76% had mutations on exon 14, and 31.43% had mutations on exon 17. All secondary *KIT* mutations were missense mutations, mostly located in the kinase domain (31). Zhao et al. obtained consistent results in an analysis of the distribution of the most common Kit mutation forms in 2,273 Chinese patients with GIST. The results showed that KIT exon 13 V654A and exon 17 N822K were the most common secondary mutations in GISTs with primary mutations in exon 11 (32). These two secondary *KIT* mutations induce resistance to IM by activating the PI3K/AKT/mTOR pathway (33). Inhibition of PI3K induces massive apoptosis in IM-resistant GISTs (34). Interestingly, *KIT* is overactivated in IM-resistant GISTs with secondary *KIT* mutations; however, the expression levels are not significantly increased. Secondary *PDGFRA* mutations are mostly located in exon 18 (24, 35).

In addition to secondary *KIT* and *PDGFRA* mutations, several additional genetic mutations have been associated with secondary resistance to IM in GISTs. Additional mutations of *RB1*, *SMARCB1*, and *MAX* (myc-related protein) are important causes of resistance to IM. Notably, GISTs caused by different gene mutations show different clinicopathological characteristics (31). Genome-scale CRISPR-Cas9 knockout (GeCKO) screening classifies *TP53* and *SOCS6* as candidate genes for resistance to IM owing to their presence in multiple signaling pathways, such as the apoptosis pathway, Wnt signaling pathway, and JAK-STAT signaling access (36).

Identifying the abovementioned types of gene mutation is a key supplement to the existing GIST risk assessment model. In addition, the discovery of new potential candidate therapeutic targets for different genetic mutations will be beneficial in delaying the progression of GISTs. Individualized therapy based on the identification of types of genetic mutation will also provide new adjuvant treatment options for patients with IM-resistant GIST. Furthermore, the type of gene mutation may be used as a biomarker to help identify patients who can benefit more from adjuvant therapy and to predict the risk of recurrence of GISTs.

### 2.2 Abnormal energy metabolism and resistance to imatinib

An important feature of cancer cells is abnormal energy metabolism, which is characterized by strong aerobic glycolysis and reduced mitochondrial energy metabolism. This feature is called the Warburg effect (37). Metabolic reprogramming of cancer cells sets the stage for rapid growth and metastasis (38, 39). Drug-resistant cancer cell subsets depend on the enhancement of mitochondrial function and OXPHOS (40, 41). Moreover, the metabolic adaptation of cancer cells to the toxic effects of targeted drugs contributes to drug resistance (42–45). GIST cells exhibit high levels of glucose uptake and aerobic glycolytic activity, and metabolic reprogramming induced by IM stress enhances mitochondrial function and OXPHOS (46).

IM alters the metabolic phenotype of GISTs (46) and increases the expression of several OXPHOS proteins, including complexes II, III, and V (40). Huang et al. found that IM-resistant GIST cells show increased OXPHOS protein expression compared with IM-sensitive GIST cells (**Figure 1A**) (40). In addition, IM-resistant GIST cells show higher OXPHOS levels and glycolysis rates than IM-sensitive cells and are more susceptible to glycolysis inhibition. Inhibition of OXPHOS increases the sensitivity of GISTs to IM. OXPHOS protein expression is increased in IM-sensitive GIST cells after IM treatment but not in IM-resistant GIST cells (47). Notably, there is a heterogeneity of metabolic phenotypes in IM-resistant GIST (40). Glucose transporter 1 (GLUT-1) is a key component of the glycolytic pathway and is associated with secondary resistance to IM in GIST cells. IM downregulates the expression of GLUT-1 and the glycolytic pathway components hexokinase 2, pyruvate kinase M2, and lactate dehydrogenase in IM-sensitive GIST cell lines. In contrast, the expression of GLUT-1 and these glycolytic pathway components increases after the treatment of IM-resistant GIST cell lines using IM (**Figure 1B**). This indicates that IM-resistant GIST cells have a higher glycolysis rate than IM-sensitive GIST cells (48).

Following chronic IM induction, energy metabolism in GIST cells shifts from the tricarboxylic acid cycle to the pentose phosphate pathway (PPP) (47). On one hand, the expression of phosphate glucose dehydrogenase (PGD), one of the rate-limiting enzymes of the PPP, is significantly upregulated in IM-resistant GIST cell lines. Overexpression of PGD promotes GIST cell proliferation and inhibits cell apoptosis. On the other hand, the level of hypoxia-inducible factor 1α (HIF-1α) is elevated under prolonged stimulation of reactive oxygen species generated by IM (47). HIF-1α leads to changes in metabolic pathways as follows: the HIF-1α–PGD–PPP axis stimulates GIST cells from the G1 phase to the S phase, inhibits GIST cell apoptosis through metabolic reprogramming, and ultimately leads to IM resistance (**Figure 1C**) (47).

Most of the research viewpoints on energy metabolism in GIST cells have reached a consensus, which provides a theoretical basis for overcoming resistance to IM from the perspective of abnormal energy metabolism. Therapy involving the inhibition of the energy metabolism pathway combined with IM, such as VLX600 combined with IM and WZB117 combined with IM, requires further preclinical validation (46, 48).

### 2.3 Non-coding RNAs and resistance to imatinib

#### 2.3.1 Long non-coding RNAs

Long non-coding RNAs (lncRNAs) are transcripts longer than 200 nucleotides with no or limited protein-coding capacity (49–51). LncRNAs play key roles in several important biological processes, including regulation of epigene expression, as well as transcriptional and posttranscriptional regulation (52). Numerous studies have demonstrated that lncRNAs play key regulatory roles in the disease course of human cancers, including cancer cell proliferation, apoptosis, and drug resistance (53–55). In addition, recent studies have shown that lncRNAs can modulate the sensitivity of patients to anticancer drugs and thus have the potential to be therapeutic targets in the treatment of drug-resistant tumors (56, 57). Moreover, lncRNAs may promote the progression and metastasis of GISTs, and the expression of many lncRNAs in primary GIST tissue differs from that in recurrent GIST tissue (58, 59). Furthermore, lncRNAs are associated with secondary resistance to IM in GISTs, and the resistance mechanisms are mostly related to signaling pathways (60–63). LncRNAs, such as the HOX antisense intergenic RNA (HOTAIR), can also promote IM resistance by activating autophagy in GIST cells (62).

**Figure 2:**
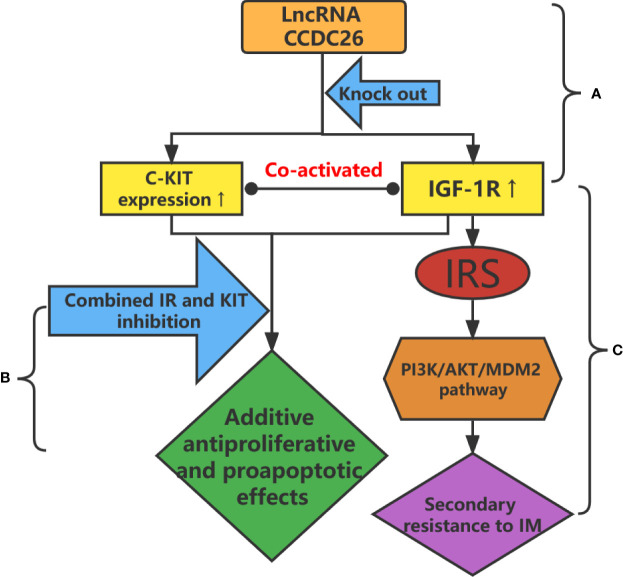
**(A)** *CCDC26* knockout upregulates the expression of C-KIT and IGF-1R in IM-resistant GISTs. **(B)** Additive antiproliferative and proapoptotic effects are obtained after the combined inhibition of IR and KIT in IM-resistant GIST cells. **(C)** Upregulation of IGF-1R leads to drug resistance through the PI3K/AKT/MDM2 signaling pathway.

The lncRNA coiled-coil domain-containing 26 (*CCDC26*), located on chromosome 8q24.21, is a retinoic acid–dependent regulator of myeloid differentiation, also known as RAM (64). *CCDC26* interacts with C-KIT and regulates its transcription. In addition, *CCDC26* downregulates the expression of c-Kit in GISTs, whereas *CCDC26* knockout induces IM resistance in GIST cells by upregulating the expression of C-KIT (**Figure 2A**) (60). *CCDC26* knockout also upregulates the expression of insulin-like growth factor 1 receptor (*IGF-1R*) (**Figure 2A**). *IGF-1R* induces drug resistance by participating in the apoptosis pathway (**Figure 2C**), whereas inhibition of *IGF-1R* reverses *CCDC26* knockout–induced drug resistance (59, 65, 66). These findings suggest that treatment targeting the CCDC26 or CCDC26-IGF-1R axis may improve sensitivity to IM in patients with IM-resistant GISTs.

Yan et al. identified a set of dysregulated lncRNAs in IM-resistant GISTs using chip technology and found that lncRNAJC6-2 is associated with the HIF-1α pathway, which links lncRNAs to energy metabolism (61). Using high-throughput RNA sequencing, Shao et al. found that GIST samples express 40% of all annotated lncRNAs in humans. Notably, the number of downregulated lncRNA expressions was greater than the number of upregulated expressions, irrespective of the presence or absence of resistance to IM. The expression of RP11616M22.7 is significantly increased in IM-resistant samples than that in non-resistant samples and is closely related to the Hippo pathway. Overexpression of RP11-616M22.7 induces resistance to IM in GIST cells, whereas RP11616M22.7 gene knockout enhances IM resistance in GIST cells both *in vitro* and *in vivo* (62).

Some studies have demonstrated that the expression and dysregulation of lncRNAs are more cancer-specific than those of protein-coding genes (67). Therefore, specific lncRNAs in GISTs are likely to be involved in unique biological functions related to treatment and drug resistance. With an in-depth exploration of specific lncRNAs and their mechanisms, our understanding of the non-coding transcriptome of GISTs will become more comprehensive, which will, in turn, accelerate the development of new effective therapeutic targets.

#### 2.3.2. MicroRNAs

**Figure 3:**
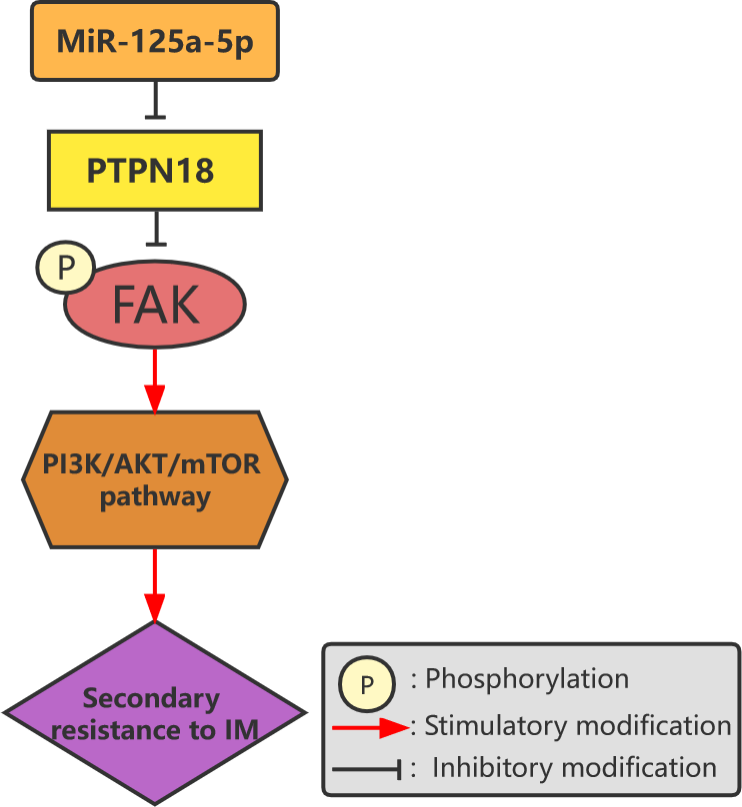
Overexpression of miR-125a-5p downregulates the expression of *PTPN18* and promotes IM resistance in GISTs mediated by phosphorylated FAK levels.

MicroRNAs (MiRNAs) are 22-nucleotide non-coding small ribonucleic acids that control tumor cell growth by regulating the expression of multiple gene products and the function of cellular pathways (68). MiRNAs play important roles in the pathogenesis, invasion, and drug resistance of tumors and are thus identified as targets for cancer diagnosis, therapy, and prognosis (69–72). Akçakaya et al. analyzed miRNA expression profiles to study the miRNA expression signatures associated with response to IM and *KIT* mutation status in patients with GIST. They found that miR-125a-5p and its target gene, tyrosine-protein phosphatase non-receptor type 18 (*PTPN18*), play important roles in IM resistance. The mechanism behind this is that overexpression of miR-125a-5p downregulates the level of *PTPN18* expression in GISTs and promotes resistance to IM (73). Subsequent studies demonstrated that the effects of miR-125a-5p and *PTPN18* on IM resistance are mediated through phosphorylated FAK levels (**Figure 3**) (28). By comparing two groups of IM-resistant GIST samples with and without secondary mutations, Amirnasr et al. detected 22 significantly differentially expressed miRNAs and almost completely separated the two groups of samples. Three of these miRNAs, namely, miR-92a-3p, miR-99a-5p, and miR-101-3p, are potential effectors of IM resistance. This suggests that the distribution of miRNA biomarkers may be related to the presence of secondary mutations (74). Zhang et al. used the microarray data preserved by Akçakaya et al. to identify five key miRNAs in the lncRNA–miRNA target gene regulatory network, confirming that overexpression of miR-28-5p and miR-125a-5p is significantly related to secondary resistance to IM (75). Kou et al. studied the miRNA expression profiles in the serums of patients with GIST and found that the levels of miR-518e-5p and miR-548e in the serums of the patients in the IM-resistant group were significantly higher than those of the patients in the IM-sensitive and healthy control groups. This indicates that the serum level of miR-518e-5p can distinguish IM-resistant patients from IM-sensitive patients or healthy individuals (76).

Studies have demonstrated that miRNAs can regulate resistance to chemotherapy by inducing autophagy in GIST cells (77, 78). Chen et al. found that miR-30a sensitizes GIST cells to IM by inhibiting autophagy and confirmed that the autophagy marker Beclin-1 is a target gene of miR-30a (79). Zhang et al. found that HOTAIR targeting the autophagy-related protein 2 homolog B inhibitor miR-130a promotes resistance to IM by upregulating the level of autophagy (63).

Information regarding most miRNAs associated with secondary resistance to IM is still in the discovery stage; thus, the resistance mechanisms need to be studied further. Because miRNAs are closely related to the pathogenesis, invasion, metastasis, and drug resistance of tumors, research ideas should be broadened rather than limited to one aspect. Several studies have confirmed that lncRNAs can regulate other non-coding RNAs, especially miRNAs, and that miRNAs also have regulatory effects on lncRNAs (80, 81). Therefore, improving the regulatory network of miRNAs and lncRNAs in IM-resistant GISTs is also a promising research direction.

### 2.4. Several key proteins and resistance to imatinib

From a protein perspective, approximately 10% of *KIT*-positive GISTs lose the expression of *KIT* oncoproteins and become resistant to TKIs owing to the transition to a *KIT*-independent state (*KIT*-negative) during TKI treatment (82). Tu et al. found that Axl in TKIs is highly expressed in KIT-negative GISTs and that *Axl* gene knockout or silencing can inhibit the proliferation of *KIT*-negative GISTs. This information provides a new perspective regarding the Axl/P53 signaling axis as a therapeutic target for a subset of *KIT*-negative GISTs (83).

Cyclin D1 can regulate the cell cycle through the activation of the cyclin-dependent kinase (CDK), activation of transcription factors, *RAD51* co-regulation of DNA repair, and activation of the AMPK-LKB1 signaling pathway (84). Cyclin D1 is highly expressed in each *KIT*-independent GIST cell subline. In addition, inhibition of cyclin D1 has antiproliferative and proapoptotic effects in *KIT*-independent GISTs, which are associated with Rb activation and p27 upregulation. Notably, *PRKCQ* is a negative regulator of cyclin D1 expression, whereas the Jun and Hippo pathway effector molecules YAP and TAZ are positive regulators of cyclin D1 expression. The *PRKCQ*, Jun, and Hippo pathways synergistically regulate cyclin D1 expression in GISTs (85). Using GeCKO screening, (85) found that *CDK1* is highly expressed in advanced and IM-resistant GISTs in three patient cohorts. *CDK1* is the founding member of the CDK family (86). It can promote the proliferation and progression of GISTs by binding to substrate protein kinase B (Akt) and regulating its phosphorylation (87). In most solid tumors, Aurora kinase A (*AURKA*) promotes cell cycle progression by regulating cell cycle checkpoints (88). A clinical analysis has demonstrated that *AURKA* can be an independent prognostic factor for GISTs. In addition, experiments have shown that overexpression of *AURKA* can promote the proliferation of GIST-T1 cells, inhibit cell apoptosis, and enhance the resistance of cells to IM (89).

Several multidrug transporters play key roles in secondary drug resistance by regulating drug concentrations in tumor cells. Multidrug resistance–related protein 1 (MRP1) is one of the major multidrug transporters (90). Intracellular IM level plays an important role in the development of IM resistance in patients with chronic myeloid leukemia (91). Studies have confirmed that MRP1 and breast cancer resistance protein are highly expressed in IM-resistant GIST cell lines and that IM-resistant patients with GIST show significantly lower intracellular IM levels than IM-sensitive patients (92). This suggests that drug transporters may play an important role in IM resistance. Xu et al. proposed the following mechanism for this: the methyltransferase *METTL3* mediates 6-methyladenosine (M6A) to modify the 5’end non-coding region of the multidrug transporter MRP1 mRNA and promotes the translation of MRP1 mRNA, leading to drug resistance in GISTs (93). M6A is a common mRNA modification that regulates mRNA stability, splicing, and translation (94, 95). These findings suggest that drug transporters may be potential therapeutic targets in the treatment of IM-resistant GISTs.

The insulin receptor (IR) is a member of the tyrosine kinase family, including homologous types 1 and 2 (*IGF-1R* and *IGF-2R*) (96). IR and *IGF-1*/*2R* play important roles in energy metabolism and cell growth, division, and differentiation (97). Chen et al. showed that IR and Kit are co-activated in IM-resistant GIST cells and biopsy samples but not in IM-sensitive GIST cells (**Figure 2A**). They also found that additive antiproliferative and proapoptotic effects were obtained after the combined inhibition of IR and *KIT* in IM-resistant GIST cells (**Figure 2B**) (98). Thus, the inactivation of IR increases the sensitivity of resistant cells to IM, suggesting that the combined inhibition of IR and *KIT* is a promising therapeutic strategy in the treatment of IM-resistant GISTs.

Serrano-Candelas et al. found that the linker molecule SH3-binding protein 2 (*SH3BP2*) is expressed in non–wild-type GISTs. *SH3BP2* is involved in the regulation of the expression and cellular activity of *KIT* and *PDGFRA* in GISTs. They also found that silencing of *SH3BP2* is accompanied by downregulation of oncogenic *KIT* and *PDGFRA* and significant promotion of apoptosis in IM-sensitive and resistant GIST cells (99).

The relationship between various key proteins and IM resistance mechanisms is intricate and interconnected. However, there is no clear comparison of the role of each protein network in the mechanism of resistance to IM. Targeted therapy that involves a single protein network may not solve the problem of secondary resistance to IM. The combined inhibition of multiple protein networks may become a new research direction for the treatment of IM-resistant GISTs.

### 2.5. Mutation and other gene aberrations and resistance to imatinib

#### 2.5.1. Oncogenic *KIT* signaling on the Golgi apparatus

**Figure 4:**
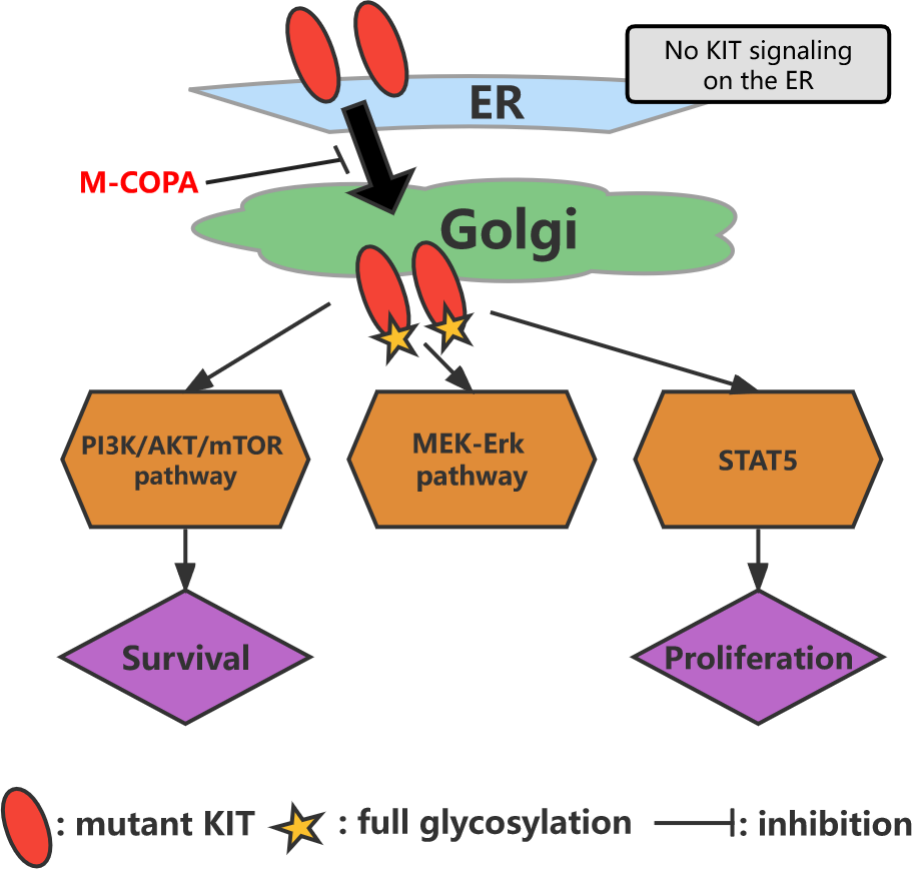
Model of oncogenic *KIT* signaling on intracellular compartments in GISTs. *KIT* is normally transported from the endoplasmic reticulum to the Golgi apparatus, followed by full glycosylation. After reacting with the Golgi apparatus, *KIT* can activate the PI3K/AKT/mTOR pathway, MEK-Erk pathway, and *STAT5*. M-COPA inhibits oncogenic signaling by blocking the transport of *KIT* from the endoplasmic reticulum to the Golgi because *KIT* activates downstream molecules only on the Golgi apparatus.

The Golgi apparatus may serve as a platform for oncogenic *KIT* signaling (100, 101). Moreover, oncogenic *KIT* signaling on the Golgi apparatus is essential for the autonomous proliferation of GIST cells (101). In IM-resistant GISTs with secondary *KIT* mutations, oncogenic *KIT* signaling is predominantly localized to the Golgi apparatus (100). This KIT activates the PI3K/AKT/mTOR pathway, MEK-Erk pathway, and signal transducer and activator of transcription 5 (**Figure 4**) (100). Blocking KIT biosynthetic transport from the endoplasmic reticulum to the Golgi apparatus suppresses oncogenic signaling, suggesting that Kit autophosphorylation is spatiotemporally regulated (100, 101). In an analysis of this mechanism, Obata et al. discovered a biosynthetic protein, 2-methylcopropylamide (M-COPA; also known as “AMF-26”), which blocks the transport of KIT from the endoplasmic reticulum to the Golgi apparatus by inhibiting the autophosphorylation of *KIT* at Y703/Y721/Y730/Y936 and ultimately inhibits oncogenic *KIT* signaling (**Figure 4**) (101). M-COPA inhibits the activation of Kit kinase domain mutants, thereby inhibiting the proliferation of IM-resistant GISTs (101). A novel heat shock protein 90 inhibitor, TAS-116, also inhibits the growth of drug-resistant cells and induces their apoptosis by reducing *KIT* autophosphorylation in the Golgi apparatus (102). Notably, the effect of TAS-116 has been validated in an animal study conducted using a xenograft mouse model (102).

Oncogenic *KIT* signaling on the Golgi apparatus provides new insights into not only the pathogenesis of KIT but also the treatment of IM-resistant GISTs that express mutant *KIT*. However, further studies are needed to confirm the clinical efficacy of drug therapies that target this carcinogenic signal.

#### 2.5.2. KIT^low^ cell subsets

KIT^low^ cell subsets may be a cell bank that mediates the progression and recurrence of GISTs (103). Bardsley et al. detected a precursor cell of ICCs in the stomach wall of a mouse that possesses stem cell properties, including the ability to self-renew and differentiate into mature ICCs. This ICC precursor cell–derived cell line was able to spontaneously transform to form GIST-like tumors. Notably, the expression of Kit in this ICC precursor cell was lower than that in mature ICC precursor cells (104, 105).

Inherently, IM-resistant *CD34* KIT^low^ cells are a distinct subset of GIST cells. KIT^low^ cells have stronger replication ability and clonogenic potential than KIT^High^ cell subsets. This subpopulation has tumor stem cell–like expression characteristics and behaviors and can self-renew and differentiate into IM-sensitive *CD34* KIT^High^ progeny. Notably, TKI treatment results in the enrichment of this KIT^low^ cell subset, which may be mediated by cell-associated transcription factors (OCT4 and NANOG) (103). The KIT^low^ cell subset represents a novel mechanism of primary resistance to TKIs and a targetable subpopulation in the treatment of GISTs. This provides valuable therapeutic ideas for overcoming the persistence and recurrence of GISTs after TKI therapy.

## 3 Conclusions

In this review, the findings of studies on mechanisms of secondary resistance conducted over the last 5 years are summarized from the aspects of abnormal energy metabolism, gene mutations, non-coding RNA, and key proteins. These previous studies provide theoretical support for solving the problem of the mechanism of resistance to IM. However, the available data on most drug-resistance mechanisms are still in the research stage. Further clinical studies are needed to confirm the safety and efficacy of utilizing drug-resistance mechanisms as potential therapeutic targets.

Addressing the problem of secondary resistance to IM has always been the key to improving the treatment outcomes and prognoses of patients with GISTs. Different resistance mechanisms are closely linked and interact with each other; thus, using a single resistance mechanism as a therapeutic target should be avoided. The combined inhibition of drug-resistance mechanisms with IM therapy and the combined inhibition of multiple drug-resistance mechanisms are expected to become new options in the treatment of GISTs. Implementing individualized therapy based on the identification of resistance mechanisms will provide new adjuvant treatment options for patients with IM-resistant GISTs, thereby delaying the progression of GISTs.

## Author contributions

Conceptualization: ZW. Article collection and analysis: YK, XH, PS, and QZ. Manuscript writing: XH and QZ. All authors contributed to the article and approved the submitted version.

## Funding

This work was supported by the National Natural Science Foundation of China (No. 81601692), the Technology Research from the Department of Education of Liaoning Province (No. JCZR2020013), and the 345 Talent Project of Shengjing hospital of China Medical University.

## Acknowledgments

The authors would like to thank all the reviewers who participated in the review and also Editage (www.editage.cn) for its linguistic assistance during the preparation of this article.

## Conflict of interest

The authors declare that the research was conducted in the absence of any commercial or financial relationships that could be construed as a potential conflict of interest.

## Publisher’s note

All claims expressed in this article are solely those of the authors and do not necessarily represent those of their affiliated organizations, or those of the publisher, the editors and the reviewers. Any product that may be evaluated in this article, or claim that may be made by its manufacturer, is not guaranteed or endorsed by the publisher.
